# Optimized ECC Implementation for Secure Communication between Heterogeneous IoT Devices

**DOI:** 10.3390/s150921478

**Published:** 2015-08-28

**Authors:** Leandro Marin, Marcin Piotr Pawlowski, Antonio Jara

**Affiliations:** 1 Department of Applied Mathematics, Computer Sciences Faculty, University of Murcia, Regional Campus of International Excellence “Campus Mare Nostrum”, Murcia 30100, Spain; 2 Institute of Information Systems, University of Applied Sciences Western Switzerland (HES-SO), Sierre 3690, Switzerland; E-Mail: jara@um.es; 3 Department of Information Technologies, Faculty of Physics, Astronomy and Applied Computer Science, Jagiellonian University, Krakow 30-348, Poland; 4 Research and Development Department, HOP Ubiquitous, 30562 Ceuti, Murcia, Spain

**Keywords:** Internet of Things, elliptic curve cryptography, Edwards curves, security, interoperability, cross-vendor, heterogeneous devices, IPv6, NXP/Jennic 5148, MSP430, large-scale pilots

## Abstract

The Internet of Things is integrating information systems, places, users and billions of constrained devices into one global network. This network requires secure and private means of communications. The building blocks of the Internet of Things are devices manufactured by various producers and are designed to fulfil different needs. There would be no common hardware platform that could be applied in every scenario. In such a heterogeneous environment, there is a strong need for the optimization of interoperable security. We present optimized elliptic curve Cryptography algorithms that address the security issues in the heterogeneous IoT networks. We have combined cryptographic algorithms for the NXP/Jennic 5148- and MSP430-based IoT devices and used them to created novel key negotiation protocol.

## 1. Introduction

The future will strongly benefit from emerging technologies, such as the Internet of Things (IoT) [1,2]. Billions of highly-constrained devices with limited computing capabilities and wireless communication interfaces will form a major part of the IoT [3]. The newly-formed web of things will positively impact our lives [4,5], but at the same time will introduce new security and privacy threats [6,7].

Many efforts have been taken by the research community addressing security issues in IoT networks. The Internet Engineering Task Force (IETF) Datagram Transport Layer Security for the Internet of Things (DTLS-IoT) [8] working group has been adapting the Transport Layer Security (TLS) protocol for the needs of the constrained IoT devices [9]. The recently-established Authentication and Authorization for Constrained Environments (ACE) IETF working group has focused on the definition of highly-secure and privacy-oriented standards for authorization and authentication in the IoT [10].

The IoT introduces many problems regarding the overwhelming number of the deployed devices, no maintenance times, limited battery life and limited computing capabilities of the devices. There are significant differences between IoT devices’ computing capabilities. The most constrained IoT devices have only several dozen kilobytes of ROM, a few kilobytes of RAM and a few megahertz of CPU. The more powerful devices have tens, hundreds and even thousands more resources.

In such an environment, the IoT devices need to operate robustly and to provide an adequate level of security. The security mechanisms should be constructed to work efficiently on very constrained devices with possibly the highest protection. The elliptic curves cryptography (ECC)-based solutions are ideal for such scenarios, due to the security equivalence of the Rivest, Shamir and Adleman (RSA) public-key cryptosystem. public key scheme, but with significantly smaller keys and computational requirements. The public key cryptography approach has the best applicability for the IoT because it does not require the device operator to set up any security credentials (like passwords) to be able to securely communicate. This approach requires significant human intervention during large-scale deployments of the IoT.

The fact that ECC provides a very high level of security with reduced resource requirements has attracted the research community. Since Version 1.2 of the TLS and the DTLS protocols, the support for the ECC has been introduced. The ECC has been embedded into the IEEE 802.15.4 standard using a hardware solution as presented in [11]. Furthermore, the authentication protocols for wireless sensors networks in [12,13], or [14] have been based on the ECC primitives to provide a high level of security with possibly the smallest resources requirements.

Many usage scenarios of the IoT solutions require different network topologies. We have assumed that one of the most common and cost-effective topologies will be the star or extended star topology. In such a setup, the more powerful (expensive) device will be working as a connection point to which the less powerful (cheaper) devices will be connecting. In our experimental environment, the MSP430-based devices are the end points connecting to the more powerful NXP/Jennic 5148 device. The MSP430 is 16-bit processor with 50 kB of ROM and 8 kB of RAM, and the NXP/Jennic 5148 has a 32-bit processor with 128 kB of ROM and RAM.

In this context, at first, we have optimized ECC algorithms for the NXP/Jennic 5148 processors. Then, we have designed a novel authentication and key negotiation protocol based on the Schnorr signature scheme. The new protocol has been integrated with the Extensible Authentication Protocol (EAP) for the IEEE 802.15.4 framework [15,16]. The new solution has been evaluated for a heterogeneous IoT network consisting of NXP/Jennic 5148 and MSP430 devices. The results have been compared to other EAP-based authentication and key negotiation methods.

The remainder of this paper is structured as follows: Section 2 presents the motivation behind this paper and describes the selected heterogeneous IoT topologies, the secure bootstrapping problematic and our approach. Section 3 is devoted to the theoretical presentation of the ECC optimization, followed by Section 4, where the description and discussion about the Schnorr signature scheme are presented. Section 5 presents the Schnorr signature scheme-based authentication and key negotiation protocol. In Section 6, we discuss the result that we have achieved during the research and state the benefits of our approach from the networking perspective. The paper ends with Section 7, which concludes the research presented.

## 2. Motivation

In this section, we present the motivation behind this research. We start from the definition and discussion about the heterogeneous IoT and the network topologies that will be dominating the IoT. After, we outline the problematic related to the secure bootstrapping in the IoT and our approach to it.

### 2.1. Heterogeneous Internet of Things

The IoT will consist of billions of devices that will be designed and manufactured by hundreds of different producers. The multitude of application domains of the IoT will force device customization from the manufacturers and its adaptation for particular solutions. This implies that the IoT will not be homogeneous from the perspective of the hardware platform. The heterogeneous IoT will be a combination of different processors with different computing capabilities in different application scenarios and a few standardized communication mechanisms. From this perspective, there is a clear need for a solution that is both optimal and standard compliant.

Due to its simplicity, the most common network topology in the IoT will be the star or extended-star topology. Such a communication setup is simple to organize, maintain and is currently one of the most commonly used. Most of our networks are currently based on the star topology, and ongoing deployments are also being based on this topology. In the following, we present two star topology-based setups that are common in many IoT scenarios.

**Figure 1:**
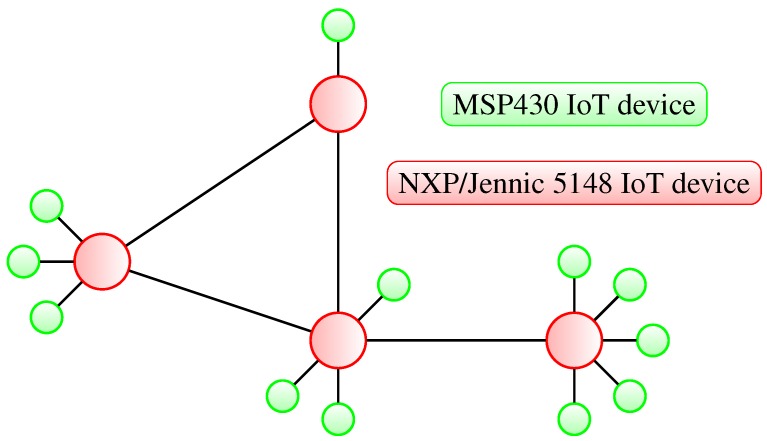
Extended star topology representation of a small heterogeneous IoT network with MSP430- and NXP/Jennic 5148-based IoT devices.

In Figure 1, we have presented a simple, star topology-based, small heterogeneous IoT network. The example network consists of two types of nodes: the first type is an MSP430-based highly constrained IoT device, and second type is a more powerful NXP/Jennic-based IoT device. The nodes are arranged in two types of topological connections. The MSP430-based devices, due to their low computational resource availability, are forming an extended star topology by connecting to the more powerful NXP/Jennic 5148 devices. The NXP/Jennic 5148 nodes are responsible for more resource-hungry tasks, like higher level device authentication or routing of the traffic between the MSP430 nodes and other parts of the network. This setup is very favorable, due to the offloading of the specific tasks from the MSP430-based devices and moving more resource-demanding jobs to the NXP/Jennic 5148 nodes. This scenario limits the deployment costs and battery usage of the more constrained devices.

**Figure 2:**
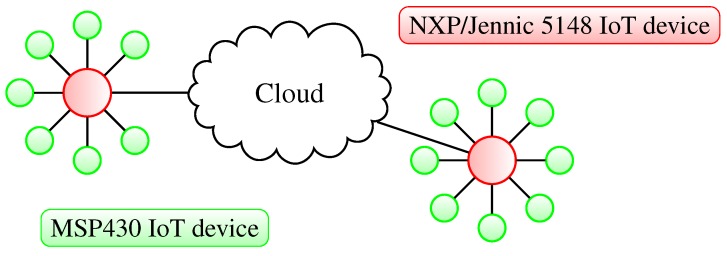
Star topology representation of small heterogeneous IoT networks comprised of MSP430- and NXP/Jennic 5148-based devices connecting directly to the cloud.

In Figure 2, a somewhat different star topology example is presented. Two separated IoT networks are connecting to each other through the cloud. The NXP/Jennic 5148 nodes are responsible for managing the connections with the MSP430 nodes and the communication with the cloud. It is highly probable that this kind of the communication scenario will dominate IoT deployments, due to the fact that the star topology is the main topology for Bluetooth Low Energy devices. We are also assuming that the large part of the new IoT devices will be based on the Bluetooth Low Energy standard.

Both example topologies will increase the operational time of the more constrained IoT devices, due to the fact that the MSP430-based devices will be able to make less computations and put themselves more often into sleep mode, thus saving energy resources. Additionally, the solutions based on star topologies with heterogeneous devices benefit from shorter latency and a smaller number of lost packets. This is related to the fact that most of the communication efforts have been shifted to the more powerful NXP/Jennic 5148 nodes, and the weaker MSP430 will not create communication delays. Using the heterogeneous approach brings down the overall hardware costs without loss of the functionality or the performance of the whole solution.

### 2.2. Secure Bootstrapping

One of the most basic functionalities that is required to create scalable IoT deployments is the secure bootstrapping mechanism. The bootstrapping process is responsible for providing necessary information for the new devices to grant them full access to the resources of the network to which they are trying to connect. In terms of the overwhelming number of IoT devices that will be deployed in the future and the constraints coming from the IoT technologies, there is an urgent need to design solutions that will enable secure bootstrapping in such an environment. Due to the fact that the network and its devices will be embedded in many scenarios that would involve gathering, sending and processing sensitive (in terms of privacy) information, the highest effort needs to be put toward providing a scalable and secure bootstrapping mechanism.

The bootstrapping process consists of a few stages, of which, from the perspective of the usage of cryptographic functionalities, the authentication process is the most important. The common way to provide the authentication in today’s networks is achieved by employment of the Extensible Authentication Protocol (EAP), which has been adapted for the IEEE 802.15.4-based IoT devices [15]. The EAP protocol provides a common framework for the authentication mechanism and can utilize many different cryptographic functions for authentication purposes. The EAP protocol works on the link layer, so it does not introduce unnecessary communication overhead and is very flexible and minimalistic. These features are very appealing for constrained devices, where every transmitted bit counts. The Transport Layer Security and Datagram Transport Layer Security are different authentication protocols that are working on a higher network layer and sharing the same feature of combining different cryptographic primitives into one solution. However, they introduce communication overhead while using the Internet Protocol version 6 (IPv6), Transmission Control Protocol (TCP) and User Datagram Protocol (UDP) protocols.

From the perspective of the IoT requirements, the best way to provide secure bootstrapping is by a combination of simple and flexible link-layer authentication protocols with a lightweight cryptographic mechanism for the authentication procedure. Such attempts have already been made and presented in [12,13] or [14]. The novelty of this research effort is that the highly-optimized elliptic curve-based authentication and key negotiation mechanisms have been combined with a very lightweight authentication protocol.

## 3. Optimization of ECC Implementation

In this section, we present the approach that has been conducted to optimize the implementation of the elliptic curve cryptography (ECC) primitives and protocols. These optimizations were especially designed for the NXP/Jennic 5148-based IoT devices and have been based on the previous work for MSP430 processors [17]. This section is theoretical and presents our approach from the mathematical point of view.

### 3.1. Point Arithmetic for ECC

ECC is based on the use of points in special curves to represent information. These curves are in the projective plane defined over a finite field. The most traditional choice is the Weierstrass curves. Despite the fact that the Weierstrass curves have a very rich literature, we will use the alternative twisted Edwards curves.

There are two reasons for this choice. The first one is to show that there are alternatives to Weierstrass curves, and the second one is that the arithmetic is better optimized with the formulas given for twisted Edwards curves. This second reason will be explained in more detail in Section 3.2.

Edwards curves were introduced in [18] and have been used in efficient cryptographical systems. These curves were generalized in [19] to the twisted Edwards curves, which are the ones that will be used in this paper.

A twisted Edwards curve is a projective curve given by the formula:

(1) $$ax^{2}+y^{2}=1+dx^{2}y^{2}$$

where *a* and *d* are the parameters of the curve. In our case, we are going to use the projective plane generated by the field $F_{p}$ for the shifting prime $p=200\cdot 256^{19}-1$ and the parameters a=92 and d=88. This is the curve given in [17].

The points of the curve can be written with two coordinates (x,y); this is what is called the affine representation. These points have a group arithmetic that can be given by unified formulas (the same for point addition and point doubling). If $P=(x_{1},y_{1})$, $Q=(x_{2},y_{2})$, then the point addition $P+Q=(x_{3},y_{3})$ has the following coordinates:

(2) $$x_{3}=\frac{x_{1}y_{2}+y_{1}x_{2}}{1+dx_{1}x_{2}y_{1}y_{2}}\;y_{3}=\frac{y_{1}y_{2}-ax_{1}x_{2}}{1-dx_{1}x_{2}y_{1}y_{2}}$$

There are several special representations for fast computation. We can give the general reference [20], where a rather complete list of formulas is provided.

If (x,y) is a point in the curve, then the points (±x,±y) are also in the curve. This implies that the number of points in the curve is a multiple of four. A subgroup of prime order is taken. We will take the same subgroup given in [17] that is generated by the point $G=(x_{G},y_{G})$ with the values:

(3) $$x_{G}=0x1898e48d78\mathrm{ff}84b21e5e96205b4f6\mathrm{bc}0b287\mathrm{caee}$$

(4) $$y_{G}=0x7\mathrm{cd}368e5542\mathrm{aa}7f0a6e3199926c006d0b47251b6$$

The number of points in this subgroup is:

(5) n=0x31fffffffffffffffffff2b53704494b52ef4695

The situation in our devices is rather different. MSP430 is a processor with very limited resources; therefore, we will try to use the best options for this processor. The implementation given in [17] by the same authors is already very optimized, and we will use it in this case. This paper will mainly consider how to connect this implementation with NXP/Jennic 5148. This second device has more computational resources, and we will delegate the computational effort to it whenever possible.

#### 3.1.1. Point Representation

The points in the curve are part of the cryptographic protocols. They can be represented by the two coordinates (x,y), but we will use only the *y*-coordinate in order to reduce the memory usage and especially the transmission time. This decision causes some problems that will be solved in Section 3.1.3.

The computation of inverses will be avoided whenever possible. All of our algorithms will give the option of having outputs in the form (A,B) representing the quotient A/B if we can use the values *A* and *B* to continue the computation and delay the computation of inverses.

#### 3.1.2. Scalar Multiplication

The main operation that is required in ECC primitives is the scalar multiplication $\left[k\right]P=\;\underset{\underset{k\;times}{︸}}{P+P+\cdots +P}$. This operation is used by almost all ECC protocols and requires the biggest part of the computational effort. The implementation of [k]P is made with a combination of point additions and doublings (the addition of a point with itself) depending on the values of the bits of *k*. This is a well-known security threat because a power analysis can detect the differences between these operations and get the value of *k* (that is, a secret value, usually the private key). It is a nontrivial problem to hide this behavior in order to increase the security.

For MSP430, we will use the implementation given in [17] that uses the formulas given in [21] for the curve arithmetic (point addition and point doubling). These formulas are especially well suited for the multiplication algorithm designed in [17] because they let one reuse the precomputations in several multiplications. The combination of point additions and point doublings to get the scalar multiplication [k]P is done with a combination of additions and subtractions that hide the actual value of *k*.

For NXP/Jennic5148, we will use the Montgomery ladder technique for twisted Edwards curves given in [22]. This method is more robust than the one implemented for MSP430, but it also requires more computational effort. This extra computational effort will be compensated with a more optimized arithmetic explained in Section 3.1.

The formulas given in [22] are prepared for curves where the parameter α=a/d is small. In that case, the testing was done with a curve in which this parameter is α=−22, but the values *a* and *d* are very big:

(6) a=0x4e42c8590b21642c8590b21642c8590b21642c89

(7) d=0x4e42c8590b21642c8590b21642c8590b21642c85

In this study, we want to use small a=92 and d=88 parameters. These parameters induce a big value of the *a* and *d* parameters with a very big *α* parameter:

(8) $$\alpha =\frac{92}{88}=0x1b45d1745d1745d1745d1745d1745d1745d1745e$$

Reusing the formulas given in [22] with a very big value of *α*, like 92/88, would require two extra multiplications. This would have a big negative impact on the performance of the implementation; therefore, we are going to provide new formulas that will solve this problem.

Following the notation given in [22], the computation requires the values $R_{\neg k_{i}}$, $S_{\neg k_{i}}$, $R_{k_{i}}$ and $S_{k_{i}}$. The values $R_{k_{i}}$ and $S_{k_{i}}$ have a problem of inefficient calculation due to the usage of the big *α* parameter.

The values $R_{t}$ and $S_{t}$ are the numerator and denominator of the actual value $\frac{R_{t}}{S_{t}}$ that is recovered at the end of the algorithm. These values are used in this form to avoid the inversion algorithm, but we can change the values $R_{t}$ and $S_{t}$ by any multiple of them $\beta R_{t}$ and $\beta S_{t}$ if the same nonzero value is used in the numerator and the denominator. The values $R_{k_{i}}$ and $S_{k_{i}}$ have to satisfy the following equation:

(9) $$\begin{array}{ccc} \frac{R_{k_{i}}}{S_{k_{i}}} & \leftarrow & \frac{R_{k_{i}}^{4}-2\alpha R_{k_{i}}^{2}S_{k_{i}}^{2}+\alpha S_{k_{i}}^{4}}{-R_{k_{i}}^{4}+2R_{k_{i}}^{2}S_{k_{i}}^{2}-\alpha S_{k_{i}}^{4}} \end{array}$$

(10) $$\begin{array}{ccc} & = & \frac{R_{k_{i}}^{4}-2\frac{92}{88}R_{k_{i}}^{2}S_{k_{i}}^{2}+\frac{92}{88}S_{k_{i}}^{4}}{-R_{k_{i}}^{4}+2R_{k_{i}}^{2}S_{k_{i}}^{2}-\frac{92}{88}S_{k_{i}}^{4}} \end{array}$$

(11) $$\begin{array}{ccc} & = & \frac{88R_{k_{i}}^{4}-92\cdot 2R_{k_{i}}^{2}S_{k_{i}}^{2}+92S_{k_{i}}^{4}}{-88R_{k_{i}}^{4}+88\cdot 2R_{k_{i}}^{2}S_{k_{i}}^{2}-92S_{k_{i}}^{4}} \end{array}$$

(12) $$\begin{array}{ccc} & = & \frac{-22R_{k_{i}}^{4}+23\cdot 2R_{k_{i}}^{2}S_{k_{i}}^{2}-23S_{k_{i}}^{4}}{22R_{k_{i}}^{4}-22\cdot 2R_{k_{i}}^{2}S_{k_{i}}^{2}+23S_{k_{i}}^{4}} \end{array}$$

The last change is done multiplying the numerator and denominator by ${\left(-4\right)}^{-1}$. The values 22 and 23 are very close. Thereby, we reuse the multiplication by 22 to the the multiplication by 23 and reorder the operations to get the result in a more effective way. The multiplication by the small constant *α* in the previous algorithm required the hidden use of an extra temporal variable. In this case, the multiplication by small constants is written in the same algorithm (Steps 23 to 29) with the temporal variable $T_{2}$.

The result is more effective than the original formulas, although the number of multiplications and squarings are the same. The optimizations do not have a big impact on the final performance, but they let us use the parameters a=92 and d=88 that were used in [17]. The final result is given in Algorithm 1.

#### 3.1.3. Recovering the *x*-Coordinate

The points of the elliptic curve have two affine coordinates (x,y), but we will represent them only with the *y*-coordinate. The *x*-coordinate of the point is needed in some cases, one of which is signature verification.

If (x,y) is a point in the curve, (−x,y) is also a point in the curve; therefore, the value of *x* is not unique, but the point should satisfy the equation $ax^{2}+y^{2}=1+dx^{2}y^{2}$; therefore, $x^{2}=\frac{1-y^{2}}{a-dy^{2}}$, and then, $x=\pm \sqrt{\frac{1-y^{2}}{a-dy^{2}}}$.

Our field is $F_{p}$ with $p=200\cdot 256^{19}-1=25\cdot 2^{155}-1\equiv 3\left(mod\;4\right)$. This kind of fields lets us compute square roots with $\pm \sqrt{m}=\pm m^{\frac{p+1}{4}}$; then, we can recover the two possibilities for *x* as:

(13) $$x=\pm {\left(\frac{1-y^{2}}{a-dy^{2}}\right)}^{25\cdot 2^{153}}$$

In our case, the value *y* will be given as $U_{0}/V_{0}$; therefore:

(14) $$\frac{1-y^{2}}{a-dy^{2}}=\frac{1-{\left(U_{0}/V_{0}\right)}^{2}}{92-88{\left(U_{0}/V_{0}\right)}^{2}}=\frac{V_{0}^{2}-U_{0}^{2}}{92V_{0}^{2}-88U_{0}^{2}}$$

This computation is given in Algorithm 2.
**Algorithm 1** Scalar multiplication (Montgomery’s technique) [a = 92, d = 88].**Input:**
P=(x,y) is a point in the curve; the order of *P* is an odd prime; $k={\left(k_{l-1},\cdots ,k_{0}\right)}_{2}$.**Output:**y([k]P) in projective coordinates.  1: **procedure**
scalarMult(k,y) 2:   $\left(R_{0},S_{0}\right)\leftarrow \left(1,1\right)$ 3:   $\left(R_{1},S_{1}\right)\leftarrow \left(y,1\right)$ 4:   **for**
i=l−1
**downto** 0 **do** 5:     $R_{\neg k_{i}}\leftarrow R_{\neg k_{i}}*S_{k_{i}}$⊳ $R_{\neg k_{i}}S_{k_{i}}$ 6:     $T_{1}\leftarrow S_{\neg k_{i}}*R_{k_{i}}$⊳ $S_{\neg k_{i}}R_{k_{i}}$ 7:     $S_{\neg k_{i}}\leftarrow T_{1}^{2}$⊳ $S_{\neg k_{i}}^{2}R_{k_{i}}^{2}$ 8:     $T_{1}\leftarrow T_{1}+R_{\neg k_{i}}$⊳ $S_{0}R_{1}+R_{0}S_{1}$ 9:     $R_{\neg k_{i}}\leftarrow R_{\neg k_{i}}^{2}$⊳ $R_{\neg k_{i}}^{2}S_{k_{i}}^{2}$ 10:     $T_{1}\leftarrow T_{1}^{2}$⊳ ${\left(S_{0}R_{1}+R_{0}S_{1}\right)}^{2}$ 11:     $S_{\neg k_{i}}\leftarrow S_{\neg k_{i}}+R_{\neg k_{i}}$⊳ $S_{0}^{2}R_{1}^{2}+R_{0}^{2}S_{1}^{2}$ 12:     $T_{1}\leftarrow T_{1}-S_{\neg k_{i}}$⊳ $2R_{0}R_{1}S_{0}S_{1}$ 13:     $R_{\neg k_{i}}\leftarrow -y*S_{\neg k_{i}}$⊳ $-y(S_{0}^{2}R_{1}^{2}+R_{0}^{2}S_{1}^{2})$ 14:     $R_{\neg k_{i}}\leftarrow R_{\neg k_{i}}+T_{1}$⊳ $-y\left(S_{0}^{2}R_{1}^{2}+R_{0}^{2}S_{1}^{2}\right)+2R_{0}R_{1}S_{0}S_{1}$ 15:     $T_{1}\leftarrow -y*T_{1}$⊳ $-2yR_{0}R_{1}S_{0}S_{1}$ 16:     $S_{\neg k_{i}}\leftarrow S_{\neg k_{i}}+T_{1}$⊳ $S_{0}^{2}R_{1}^{2}+R_{0}^{2}S_{1}^{2}-2yR_{0}R_{1}S_{0}S_{1}$ 17:     $T_{1}\leftarrow R_{k_{i}}^{2}$⊳ $R_{k_{i}}^{2}$ 18:     $S_{k_{i}}\leftarrow S_{k_{i}}^{2}$⊳ $S_{k_{i}}^{2}$ 19:     $R_{k_{i}}\leftarrow T_{1}^{2}$⊳ $R_{k_{i}}^{4}$ 20:     $T_{1}\leftarrow T_{1}-S_{k_{i}}$⊳ $R_{k_{i}}^{2}-S_{k_{i}}^{2}$ 21:     $T_{1}\leftarrow T_{1}^{2}$⊳ $R_{k_{i}}^{4}-2R_{k_{i}}^{2}S_{k_{i}}^{2}+S_{k_{i}}^{4}$ 22:     $S_{k_{i}}\leftarrow S_{k_{i}}^{2}$⊳ $S_{k_{i}}^{4}$ 23:     $T_{2}\leftarrow 0$⊳ 0 24:     $T_{2}\leftarrow T_{2}-T_{1}$⊳ $-(R_{k_{i}}^{4}-2R_{k_{i}}^{2}S_{k_{i}}^{2}+S_{k_{i}}^{4})$ 25:     $T_{2}\leftarrow T_{2}+T_{2}$⊳ $-2(R_{k_{i}}^{4}-2R_{k_{i}}^{2}S_{k_{i}}^{2}+S_{k_{i}}^{4})$ 26:     $T_{2}\leftarrow T_{2}+T_{2}$⊳ $-4(R_{k_{i}}^{4}-2R_{k_{i}}^{2}S_{k_{i}}^{2}+S_{k_{i}}^{4})$ 27:     $T_{2}\leftarrow T_{2}-T_{1}$⊳ $-5(R_{k_{i}}^{4}-2R_{k_{i}}^{2}S_{k_{i}}^{2}+S_{k_{i}}^{4})$ 28:     $T_{2}\leftarrow T_{2}+T_{2}$⊳ $-10(R_{k_{i}}^{4}-2R_{k_{i}}^{2}S_{k_{i}}^{2}+S_{k_{i}}^{4})$ 29:     $T_{2}\leftarrow T_{2}-T_{1}$⊳ $-11(R_{k_{i}}^{4}-2R_{k_{i}}^{2}S_{k_{i}}^{2}+S_{k_{i}}^{4})$ 30:     $T_{2}\leftarrow T_{2}+T_{2}$⊳ $-22(R_{k_{i}}^{4}-2R_{k_{i}}^{2}S_{k_{i}}^{2}+S_{k_{i}}^{4})$ 31:     $S_{k_{i}}\leftarrow S_{k_{i}}-T_{2}$⊳ $22R_{k_{i}}^{4}-22\cdot 2R_{k_{i}}^{2}S_{k_{i}}^{2}+23S_{k_{i}}^{4})$ 32:     $T_{2}\leftarrow T_{2}-T_{1}$⊳ $-23(R_{k_{i}}^{4}-2R_{k_{i}}^{2}S_{k_{i}}^{2}+S_{k_{i}}^{4})$ 33:     $R_{k_{i}}\leftarrow R_{k_{i}}+T_{2}$⊳ $-22R_{k_{i}}^{4}+23\cdot 2R_{k_{i}}^{2}S_{k_{i}}^{2}-23S_{k_{i}}^{4})$ 34:   **end for** 35:   **return**
$\left(R_{0},S_{0}\right)$ or $\left(R_{0},S_{0},R_{1},S_{1}\right)$ as required.⊳ The result in affine representation is $R_{0}/S_{0}$ 36: **end procedure**
**Algorithm 2** Computing the *x*-coordinate of scalar multiplication when only the original *y*-coordinate is known. 1: **procedure**
xcoordinate($U_{0},V_{0}$) 2:   $A\leftarrow V_{0}$⊳ $V_{0}$ 3:   A←A*A⊳ $V_{0}^{2}$ 4:   $B\leftarrow U_{0}$⊳ $U_{0}$ 5:   B←B*B⊳ $U_{0}^{2}$ 6:   B←B−A⊳ $U_{0}^{2}-V_{0}^{2}$ 7:   C←B⊳ $U_{0}^{2}-V_{0}^{2}$ 8:   C←C+C⊳ $2(U_{0}^{2}-V_{0}^{2})$ 9:   C←C+C⊳ $4(U_{0}^{2}-V_{0}^{2})$ 10:   C←C+B⊳ $5(U_{0}^{2}-V_{0}^{2})$ 11:   C←C+C⊳ $10(U_{0}^{2}-V_{0}^{2})$ 12:   C←C+B⊳ $11(U_{0}^{2}-V_{0}^{2})$ 13:   C←C+C⊳ $22(U_{0}^{2}-V_{0}^{2})$ 14:   C←C−A⊳ $22U_{0}^{2}-23V_{0}^{2}$ 15:   C←C+C⊳ $44U_{0}^{2}-46V_{0}^{2}$ 16:   C←C+C⊳ $88U_{0}^{2}-92V_{0}^{2}$ 17:   $C\leftarrow C^{-1}\left(mod\;p\right)$ 18:   B←B*C 19:   **for**
i=1
**to**
153
**do** 20:     B←B*B 21:   **end for** 22:   Q←B 23:   Q←Q*Q 24:   Q←Q*B 25:   Q←Q*Q 26:   Q←Q*Q 27:   Q←Q*Q 28:   Q←Q*B⊳ ${\left(\frac{V_{0}^{2}-U_{0}^{2}}{92V_{0}^{2}-88U_{0}^{2}}\right)}^{25\cdot 2^{153}}$ 29:   **return**
*Q* 30: **end procedure**

This algorithm has two possible solutions ±x. There is another case in which we can recover the *x*-coordinate. It is after the computation of the scalar multiplication if we know the original coordinate of the point. This is the case for example when we compute the scalar multiplication [s]G for the generator *G* because the coordinates of *G* are constant in the system and can be included in the program.

Suppose P=(x,y) and *k* is an integer. The computation of [k]P in Algorithm 1 provides the values $R_{0}$,$R_{1}$ and $S_{0}$,$S_{1}$, which correspond to Proposition 2 of [22] to the values $y_{n}=\frac{R_{0}}{S_{0}}$ and $y_{n+1}=\frac{R_{1}}{S_{1}}$ (with the notations from that paper). The value x([k]P) is the value $x_{n}$ given in that formula:

(15) $$\begin{array}{ccc} x([k]P) & = & \frac{y\frac{R_{0}}{S_{0}}-\frac{R_{1}}{S_{1}}}{x\left(92-88y\frac{R_{0}}{S_{0}}\frac{R_{1}}{S_{1}}\right)} \end{array}$$

(16) $$\begin{array}{ccc} & = & \frac{yR_{0}S_{1}-R_{1}S_{0}}{x\left(88S_{0}S_{1}-92yR_{0}R_{1}\right)} \end{array}$$

This computation is developed in Algorithm 3. In this case, the result is unique. The result is given as a quotient N/4D or with the values *N* and *D* that can be used to continue the computations without the inversion of 4D. The application of this will be explained in Section 4.
**Algorithm 3** Computing the *x*-coordinate of scalar multiplication when both original coordinates are known. 1: **procedure**
xcoordinate($R_{0},S_{0},R_{1},S_{1},y,x$) 2:   $A\leftarrow S_{0}$⊳ $S_{0}$ 3:   $A\leftarrow A*S_{1}$⊳ $S_{0}S_{1}$ 4:  B←y⊳ *y* 5:   $B\leftarrow B*R_{0}$⊳ $yR_{0}$ 6:   $B\leftarrow B*R_{1}$⊳ $yR_{0}R_{1}$ 7:   D←A⊳ $S_{0}S_{1}$ 8:   D←D−B⊳ $S_{0}S_{1}-yR_{0}R_{1}$ 9:   A←D⊳ $S_{0}S_{1}-yR_{0}R_{1}$ 10:   D←D+D⊳ $2(S_{0}S_{1}-yR_{0}R_{1})$ 11:   D←D+D⊳ $4(S_{0}S_{1}-yR_{0}R_{1})$ 12:   D←D+A⊳ $5(S_{0}S_{1}-yR_{0}R_{1})$ 13:   D←D+D⊳ $10(S_{0}S_{1}-yR_{0}R_{1})$ 14:   D←D+A⊳ $11(S_{0}S_{1}-yR_{0}R_{1})$ 15:   D←D+D⊳ $22(S_{0}S_{1}-yR_{0}R_{1})$ 16:   D←D−B⊳ $22S_{0}S_{1}-23yR_{0}R_{1}$ 17:   D←D*x⊳ $x(22S_{0}S_{1}-23yR_{0}R_{1})$ 18:   N←y⊳ *y* 19:   $N\leftarrow N*R_{0}$⊳ $yR_{0}$ 20:   $N\leftarrow N*S_{1}$⊳ $yR_{0}S_{1}$ 21:   $B\leftarrow R_{1}$⊳ $R_{1}$ 22:   $B\leftarrow B*S_{0}$⊳ $R_{1}S_{0}$ 23:   N←N−B⊳ $yR_{0}S_{1}-R_{1}S_{0}$ 24:   **return**
N/4D or (N,D) as needed. 25: **end procedure**


### 3.2. Arithmetic Optimization for ECC Primitives

The primary advantage of this method comes from a specialized implementation for the algorithm of modular squaring. Our biggest effort in this paper has been the implementation of this squaring algorithm, which suits perfectly the formulas given in [22]. A special squaring function cannot be used in MSP430, because it would require almost double the size of the memory, a resource that is critical in MSP430. In that case, the formulas given in [17] with optimizations derived from the reuse of the precomputations are the best solution.

The proposed optimizations of the squaring algorithm have the biggest impact on the performance of our ECC implementation, due to the fact that in the Edward curves, the scalar multiplication is used most of the time. Thus, we have completely rewritten the squaring algorithm that theoretically is able to reduce the time of the standard multiplication by more than half.

In this implementation, we have developed a square function that is especially suited to the Jennic 5148 and the shifting primes with 160 bits of size. The technique follows these principles:

- There are no loops or counters.
- Numbers are represented in Montgomery form.
- The number of memory readings is reduced to the minimal amount, keeping everything in registers as much as possible.
- The result is built starting from the least significant values to the most significant ones, and the reduction modulo $p=200\times 256^{19}-1$ is done at the same time as the multiplications.
- The carries are avoided taking in consideration the following property: if *x* and *y* are 16-bit values, the product x*y can be added with another 16-bit value without a carry.
- All crossed products x*y and y*x are computed only once and accumulated once. The final result is multiplied by two to have this in consideration.

Using all of these techniques, the modular square requires only sixty percent of the time required for the modular multiplication.

## 4. Schnorr Signature Scheme

As a usage example of the proposed optimizations, we will use the Schnorr signature scheme. The signature will be using point representation given by the *y*-coordinate. The scheme consists of three functions: key generation, signature generation and signature verification. All of these functions will be described, and the algorithms will be presented in this section.

### 4.1. Key Generation

Keys have two parts: the public key (pk) that is a point in the elliptic curve, and we will represent it with its *y*-coordinate; and the private key (sk) that is a number in the range of *n*, the number of points in the subgroup generated by *G*. These parameters are given in Section 3.1. The key generation is given in Algorithm 4.
**Algorithm 4** Key generation.**Output:** returns (pk,sk), the public key and private key.  1: **procedure**
kg 2:   sk←rand(n)⊳ A random value in the range of *n* 3:   pk←scalarMult($sk,y_{G}$)⊳ Using Algorithm 1 or the algorithm given in [17], we compute  the *y*-coordinate of [sk]G for the generator *G* fixed in affine representation. 4:   **return**
(pk,sk). 5: **end procedure**


The computation of the scalar multiplication [sk]G is done with Algorithm 1 on NXP/Jennic 5148 or with the algorithm given in [17] for MSP430. In this case, both coordinates of *G* are required, and we can get both coordinates of [sk]G. Only the *y*-coordinate will be used as the public key.

### 4.2. Signature Generation

The signature generation is given in Algorithm 5.
**Algorithm 5** Signature generation.**Input:**sk is the private key of the signer, and *m* is the message**Output:** returns (s,h), the signature for the message *m*.  1: **procedure**
sgn((m,sk)) 2:   r←rand(n)⊳ A random value in the range of *n* 3:   R←scalarMult($r,y_{G}$) 4:   h←H(R∥m)⊳ the hash of the joined value R∥m 5:   s←r+sk·h(modn) 6:   **return**
(s,h). 7: **end procedure**


In this case, as we did in the hey generation, the scalar multiplication is done with different algorithms depending on the microprocessor.

### 4.3. Signature Verification

The standard Schnorr protocol for signature verification is given in Algorithm 6.
**Algorithm 6** Schnorr signature verification.**Input:**pk is the public key of the signer; *m* is the message, and (s,h) is the signature.**Output:** returns true if the signature is valid and false otherwise. 1: **procedure**
vfy(pk,m,s,h) 2:   R←y([s]G−[h]pk) 3:   **if**
H(R∥m)=h
**then** 4:     **return** true. 5:   **else** 6:     **return** false. 7:   **end if** 8: **end procedure**


The problem in this algorithm is the computation of the *y*-coordinate of the point [s]G−[h]pk needed in Step 2. This operation requires the computation of the points $\left[s\right]G=\underset{\underset{s\;times}{︸}}{G+G+\cdots +G}$ and $\left[h\right]pk=\;\underset{\underset{h\;times}{︸}}{pk+pk+\cdots +pk}$, and then, the difference [s]G−[h]pk. The scalar multiplication [s]G and [h]pk can be computed with the Algorithm 1; however, this algorithm only computes the *y*-coordinate, and we require both coordinates to compute [s]G−[h]pk. This can be done using Algorithm 2 in the case [k]pk and with Algorithm 3 for [s]G.

When we have both coordinates $\left[s\right]G=\left(x([s]G),y([s]G)\right)$ and $\left[h\right]pk=\left(x([h]pk),y([h]pk)\right)$, then R=y([s]G−[h]pk) is given by Equation (2) given in Section 3.1, and using the fact that −[h]pk=(−x([h]pk),y([h]pk)), we get:

(17) $$y\left(\left[s\right]G-\left[h\right]pk\right)=\frac{y([s]G)\;y([h]pk)+92\;x([s]G)\;x([h]pk)}{1+88\;x([s]G)x([h]pk)y([s]G)y([h]pk)}$$

Using the notations given by the previous algorithms, we have:

(18) $$\begin{array}{ccc} y([s]G) & = & \frac{R_{0}}{S_{0}} \end{array}$$

(19) $$\begin{array}{ccc} x([s]G) & = & \frac{y_{G}\frac{R_{0}}{S_{0}}-\frac{R_{1}}{S_{1}}}{x_{G}\left(92-88y_{G}\frac{R_{0}}{S_{0}}\frac{R_{1}}{S_{1}}\right)} \end{array}$$

(20) $$\begin{array}{ccc} & = & \frac{y_{G}R_{0}S_{1}-R_{1}S_{0}}{x_{G}\left(88S_{0}S_{1}-92y_{G}R_{0}R_{1}\right)}=\frac{N}{4D} \end{array}$$

(21) $$\begin{array}{ccc} y([h]pk) & = & \frac{U_{0}}{V_{0}} \end{array}$$

(22) $$\begin{array}{ccc} x([h]pk) & = & \pm {\left(\frac{1-y{\left(\left[h\right]pk\right)}^{2}}{92-88y{\left(\left[h\right]pk\right)}^{2}}\right)}^{25\cdot 2^{153}} \end{array}$$

(23) $$\begin{array}{ccc} & = & \pm {\left(\frac{1-{\left(U_{0}/V_{0}\right)}^{2}}{92-88{\left(U_{0}/V_{0}\right)}^{2}}\right)}^{25\cdot 2^{153}} \end{array}$$

(24) $$\begin{array}{ccc} & = & \pm {\left(\frac{V_{0}^{2}-U_{0}^{2}}{92V_{0}^{2}-88U_{0}^{2}}\right)}^{25\cdot 2^{153}}=\pm Q \end{array}$$

Step 2 in Algorithm 6 is:

(25) $$\begin{array}{ccc} R & \leftarrow & \frac{y([s]G)\;y([h]pk)+92\;x([s]G)\;x([h]pk)}{1+88\;x([s]G)x([h]pk)y([s]G)y([h]pk)} \end{array}$$

(26) $$\begin{array}{ccc} & = & \frac{\frac{R_{0}}{S_{0}}\frac{U_{0}}{V_{0}}\pm 92\frac{N}{4D}Q}{1\pm 88\frac{N}{4D}Q\frac{R_{0}}{S_{0}}\frac{U_{0}}{V_{0}}} \end{array}$$

(27) $$\begin{array}{ccc} & = & \frac{\frac{R_{0}}{S_{0}}\frac{U_{0}}{V_{0}}\pm 23\frac{N}{D}Q}{1\pm 22\frac{N}{D}Q\frac{R_{0}}{S_{0}}\frac{U_{0}}{V_{0}}} \end{array}$$

(28) $$\begin{array}{ccc} & = & \frac{DR_{0}U_{0}\pm 23NQS_{0}V_{0}}{DS_{0}V_{0}\pm 22NQR_{0}U_{0}} \end{array}$$

This has two possible solutions, and we combine them in order to get a common denominator; therefore, the computing inverses only once:

(29) $$\begin{array}{ccc} R_{pos} & \leftarrow & \frac{\left(DR_{0}U_{0}+23NQS_{0}V_{0}\right)\left(DS_{0}V_{0}-22NQR_{0}U_{0}\right)}{{\left(DS_{0}V_{0}\right)}^{2}-{\left(22NQR_{0}U_{0}\right)}^{2}} \end{array}$$

(30) $$\begin{array}{ccc} R_{neg} & \leftarrow & \frac{\left(DR_{0}U_{0}-23NQS_{0}V_{0}\right)\left(DS_{0}V_{0}+22NQR_{0}U_{0}\right)}{{\left(DS_{0}V_{0}\right)}^{2}-{\left(22NQR_{0}U_{0}\right)}^{2}} \end{array}$$

With these two values, we will compute the hash in Step 3. The complete algorithm decomposed in basic operations is given in Algorithm 7.

This signature verification is designed for the NXP/Jennic 5148, which in our case will be the one that requires this algorithm. In case we need an implementation of the signature verification for MSP430, we cannot compute [h]pk without the *x*-coordinate of the public key. Algorithm 2 is required to compute it before making the scalar multiplication. The algorithm implemented in [17] computes both coordinates of the scalar multiplications, and they are combined with Equation 17 afterwards.

### 4.4. Evaluation

Most of the time required for the computation of Schnorr signatures is used for the scalar multiplication. With the formulas that we have provided and the 160-bit key length, the time required for the scalar multiplication on Jennic 5148 is (on average) 112 ms. The final time for key generation is 135 ms, most of it used in the scalar multiplication.

The time for signature verification is double this quantity, because the algorithm is not using something similar to the Shamir trick.

The main reason for the time reduction in this Jennic 5148 implementation comes from the specialized square function and the use of an algorithm in which most of the operations are in fact squares.

In the case of MSP430, we do not use a specialized square, because it would double the size of the code, and the multiplication function is already big. The multiplication used is the one given in [17] by the same authors. The signature verification is done in this case with the Shamir trick; therefore, the time required is not double the time of signature generation, but only 1.5-times.
**Algorithm 7** Signature verification.**Input:**
pk is the public key of the signer; *m* is the message, and (s,h) is the signature.**Output:** returns true if the signature is valid and false otherwise. 1: **procedure**
vfy(pk,m,s,h) 2:   r←rand(n)⊳ A random value 3:   $R_{0},S_{0},R_{1},S_{1}\leftarrow$scalarMult$s,y_{G}$ 4:   $U_{0},V_{0}\leftarrow$scalarMult(h,pk) 5:   Q←xcoordinate($U_{0},V_{0}$) 6:   N,D←xcoordinate($R_{0},S_{0},R_{1},S_{1},y_{G},x_{G}$) 7:   A←N⊳ *N* 8:   A←A*Q⊳ NQ 9:   B←A⊳ NQ 10:   A←A+A⊳ 2NQ 11:   A←A+A⊳ 4NQ 12:   A←A+B⊳ 5NQ 13:   A←A+A⊳ 10NQ 14:   A←A+B⊳ 11NQ 15:   A←A+A⊳ 22NQ 16:   B←B+A⊳ 23NQ 17:   $A\leftarrow A*R_{0}$⊳ $22NQR_{0}$ 18:   $A\leftarrow A*U_{0}$⊳ $22NQR_{0}U_{0}$ 19:   $B\leftarrow B*S_{0}$⊳ $23NQS_{0}$ 20:   $B\leftarrow B*V_{0}$⊳ $23NQS_{0}V_{0}$ 21:   E←D⊳ *D* 22:   F←E⊳ *D* 23:   $E\leftarrow E*R_{0}$⊳ $DR_{0}$ 24:   $E\leftarrow E*U_{0}$⊳ $DR_{0}U_{0}$ 25:   $F\leftarrow F*S_{0}$⊳ $DS_{0}$ 26:   $F\leftarrow F*V_{0}$⊳ $DS_{0}V_{0}$ 27:   $R_{pos}\leftarrow E$
 28:   $R_{neg}\leftarrow E$
 29:   $R_{pos}\leftarrow R_{pos}+B$⊳ $DR_{0}U_{0}+23NQS_{0}V_{0}$ 30:   $R_{neg}\leftarrow R_{neg}-B$⊳ $DR_{0}U_{0}-23NQS_{0}V_{0}$ 31:   B←F
 32:   E←F
 33:   B←B−A⊳ $DS_{0}V_{0}-22NQR_{0}U_{0}$ 34:   E←E+A⊳ $DS_{0}V_{0}+22NQR_{0}U_{0}$ 35:   $R_{pos}\leftarrow R_{pos}*B$⊳ $\left(DR_{0}U_{0}+23NQS_{0}V_{0}\right)\left(DS_{0}V_{0}-22NQR_{0}U_{0}\right)$ 36:   $R_{neg}\leftarrow R_{neg}*E$⊳ $\left(DR_{0}U_{0}-23NQS_{0}V_{0}\right)\left(DS_{0}V_{0}+22NQR_{0}U_{0}\right)$ 37:   E←F⊳ $DS_{0}V_{0}$ 38:   E←E−A⊳ $DS_{0}V_{0}-22NQR_{0}U_{0}$ 39:   F←F+A⊳ $DS_{0}V_{0}+22NQR_{0}U_{0}$ 40:   F←F*E⊳ $\left(DS_{0}V_{0}+22NQR_{0}U_{0}\right)\left(DS_{0}V_{0}-22NQR_{0}U_{0}\right)$ 41:   $F\leftarrow F^{-1}\left(mod\;p\right)$
 42:   $R_{pos}\leftarrow R_{pos}*F$
 43:   $R_{neg}\leftarrow R_{neg}*F$
 44:   **if**
$H(R_{pos}∥m)=h$
**or**
$H(R_{neg}∥m)=h$
**then**
 45:     **return** true. 46:   **else** 47:     **return** false. 48:   **end if** 49: **end procedure**


## 5. Schnorr Authentication and Key Negotiation Protocol

**Figure 3:**
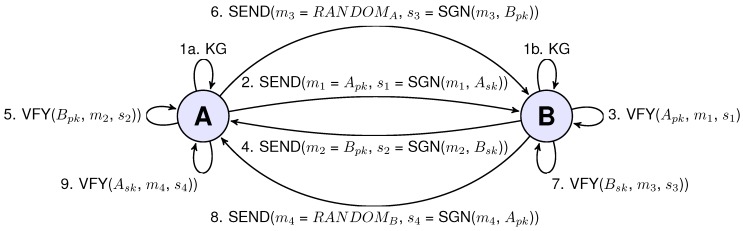
Schnorr signatures scheme-based authentication and key negotiation protocol.

We have designed a Schnorr signature scheme-based authentication and key negotiation protocol. The newly-proposed scheme consists of an exchange of four communications, and it has been presented in Figure 3. This authentication scheme has been integrated with the Extensible Authentication Protocol (EAP) that previously had been adapted for the needs of the IEEE 802.15.4 IoT-based networks [15,16]. The scheme presents the benefits of using optimized ECC primitives with shifting primes curves over different, standard curves. We will refer to this new protocol as EAP-SCHNORR, due to the nomenclature coming from the EAP protocol.

### 5.1. Protocol Description

The first step of the scheme is the key generation phase (Algorithm 4), after which, both devices will be in possession of their public and private keys, ($\left(A_{pk},A_{sk}\right)$) for Device A and ($\left(B_{pk},B_{sk}\right)$) for Device B. If the devices have generated their credentials, then Device A starts the key negotiation procedure by sending its public key $m_{1}$ = $A_{pk}$ alongside its signature $s_{1}$ = SGN($m_{1}$, $A_{sk}$). After the reception of the message sent by Device A, Device B verifies the authenticity of the message by checking its signature with Algorithm 6. After successful verification, Device B sends its public key $m_{2}$ = $B_{pk}$ alongside its signature $s_{2}$ = SGN($m_{2}$, $B_{sk}$) to Device A. After reception of the message from Device B, Device A verifies the signature of the message with Algorithm 6. Next, Device A sends the random value $RANDOM_{A}$ along with its signature generated with Device B public key ($B_{p}k$). During the signature generation with Algorithm 5, the device saves the *R* value (Line 3 of the Algorithm 5) as part of the shared key ($key_{A}$). Then, Device B receives the message from Device A and verifies the signature with its secret key. During the verification, Device B saves the *R* value (Line 2 of the Algorithm 6) as part of the shared key ($key_{A}$). After that, Device B sends random value $RANDOM_{B}$ with its signature using the Device A public key ($A_{pk}$). During the signature generation, Device B saves the *R* value as the second part of the shared secret ($key_{B}$). Then, Device A receives the message from Device B and verifies its signature with its private key ($A_{sk}$), during which Device A saves the *R* value as the second part of the shared secret ($key_{B}$). If the verification went successfully, both of the devices will be able to generate a working shared key from the two parts of the shared secret using key generation procedure ($keygen(key_{A},key_{B})$). For the simplicity of the scheme, we have assumed that the keygen() function is XORing the keys; different operations on the keys are also possible, but further deliberation on this matter is out of the scope of this paper. It should be mentioned that the output of the keygen() procedure should be design to fulfil the needs of the encryption mechanism that is going to be applied by the communicating parties.

### 5.2. Security Discussion

The security of the EAP-SCHNORR protocol lies in the Schnorr signature scheme that is based on the elliptic curve cryptography primitives that are utilizing the hardness of the elliptic curve discrete logarithm problem (ECDLP). The protocol requires exchanging four signatures, of which the first two are for the purpose of connection initiation, during which their identities (in the form of the public keys) are exchanged. The last two signature exchanges are for the verification of the identity and for the common key generation.

It is easy to notice that the parties involved in this protocol message exchange are vulnerable to the man-in-the-middle attack. This can only be mitigated for the second and following key negotiations when parties have stored theirs peers’ valid public keys. Another technique avoiding the man-in-the-middle attack needs to involve a trusted third party that would confirm the validity of the public keys.

Due to the fact that the EAP-SCHNORR protocol has been designed to show the benefits of optimized ECC primitives with shifting primes curves, the security of the protocol has not been the main objective. However, we are considering to focus more work in that area in the near future.

## 6. Results Discussion

We have performed network usage measurements using a previous implementation of the EAP protocol and its selected methods. The statistics have been extended with EAP-SCHNORR method results based on the signature-based key negotiation procedure from Section 5. The EAP-SCHNORR method results have been estimated for the heterogeneous environment consisting of MSP430- and NXP/Jennic 5148-based nodes, as described in Section 2.2. The EAP methods that are different from EAP-SCHNORR do not include any optimizations that have been described in this paper.

The EAP-SCHNORR method as the Schnorr signature scheme requires a hash function to work. Thus, we have introduced four different variations of the EAP-SCHNORR method based on four different cryptographic hash functions. We have EAP-SCHNORR method where Message Digest algorithm version 5 (MD5) [23] hash function have been used, EAP-SCHNORR with Secure Hash Algorithm version 1 (SHA1) [24] as a hash function and EAP-SCHNORR-SHA256 and EAP-SCHNORR-SHA512 based on the SHA-2 family of hash functions [25]. It is noteworthy to mention that the usage of the MD5 and SHA-1 hash functions should be depreciated due to security reasons. This hash functions have been only included in the experiment for reference purposes.

The EAP-SCHNORR message exchange procedure consists of four different communications. However, all of the communications are constructed in a very similar way. The first and second communications that are exchanged consist of the ECC public key (20 bytes) and its corresponding signature, whose length depends on the employed hash function. The third and fourth messages differ from the first two in that instead of the ECC public key, they are transmitting random values. In our case, a random value has the the same 20-byte length as the public key, but this length is not a requirement and can be set as required.

From the results presented in Table 1, it can be observed that the differences in the network resource usage in EAP-SCHNORR are mainly due to the usage of the different cryptographic hash functions. This is obviously related to the hash function output length: MD5 has a 16-byte output size; the SHA-1 has a 20-byte output size; SHA256 has a 32-byte output size; and SHA512 has a 64-byte output size.

**Table 1:** Network usage statistics of different Extensible Authentication Protocol (EAP) methods of EAP communication, calculated on the MSP430 node connecting to the NXP/Jennic 5148, during the authentication procedure. TLS, Transport Layer Security.

	TX Packets	TX Data	RX Packets	RX Data	Total Packets	Total Data
EAP-SCHNORR-MD 5	2	122 B	2	122 B	4	244 B
EAP-SCHNORR-SHA 1	2	130 B	2	130 B	4	260 B
EAP-SCHNORR-SHA256	2	154 B	2	154 B	4	308 B
EAP-SCHNORR-SHA512	2	218 B	2	218 B	4	436 B
EAP-MD5	3	66 B	3	59 B	6	125 B
EAP-PSK	5	181 B	4	160 B	9	341 B
EAP-TLS-ECDSA -160	12	271 B	17	812 B	29	1083 B
EAP-TLS-ECDSA-256	13	286 B	18	931 B	31	1217 B
EAP-TLS-RSA -480	19	376 B	24	1566 B	43	1942 B
EAP-TLS-RSA-512	20	397 B	25	1627 B	45	2024 B
EAP-TLS-RSA-1024	27	496 B	32	2370 B	59	2866 B
EAP-TLS-RSA-2048	43	712 B	48	4200 B	91	4912 B

In comparison to the other evaluated EAP methods the EAP-SCHNORR-based methods are some of the most lightweight ones. All of them, except EAP-SCHNORR-SHA512, have used the number of network resources between EAP-MD5 and EAP-PSK, with the total data used between 244 bytes (MD5 based) and 308 bytes (SHA256 based). The EAP-SCHNORR-SHA512 uses 436 bytes of network resources and has been placed between the EAP-PSK and EAP-TLS wit Elliptic Curve Digital Signature Algorithm wit 160 bit keys (EAP-TLS-ECDSA-160) methods.

The benefits of using the EAP-SCHNORR method over other methods are due to the fact that this method requires transmitting the minimal number of packets. Additionally, the EAP-SCHNORR method is based on public key cryptography, so it does not require a setup phase during which the shared key is entered into the devices. The EAP-MD5 and EAP-PSK use private key cryptography, and due to that, large-scale deployments using these methods would require much effort and maintenance time. Lastly, the security of EAP-MD5 is known to be highly defective due to the weakness of the MD5 hash function, so it is completely unwise to be designing security solutions using this method.

## 7. Conclusions

In this paper, we have presented ECC optimization for secure communication in heterogeneous Internet of Things networks. The work presented in this research has been focused on providing optimized ECC algorithms for the NXP/Jennic 5148-based IoT devices that could be utilized with MSP430-optimized counterparts for secure communication in IoT networks. The Schnorr signature scheme has been used as an exemplary solution utilizing our optimized ECC algorithms. In addition, we have designed a simple key negotiation protocol based on the Schnorr scheme that demonstrates the usability of the presented ECC optimizations.

Ongoing work is focused on the optimization of the ECC primitives for the needs of the different microprocessors, and a more advanced authentication mechanism is being designed. Effort is also put into the integration of the proposed solution with Trust Extension Protocol for Authentication of New deployed Objects and sensors through the Manufacturer (TEPANOM) [26], and also, an extension of this work is envisioned for the Bluetooth Low Energy devices for the needs of the truly heterogeneous IoT ecosystem.

## References

[1] Patrick G., Peter F. (2009). The Internet of Things—Strategic Reseach Roadmap.

[2] Union I.T. (2005). The Internet of Things—Executive Summary.

[3] Cisco about Internet of Things. http://www.eetimes.com/electronics-news/4409928/Cisco-sees–14-trillion-opportunity-in-Internet-of-Things.

[4] Taylor A.S., Harper R., Swan L., Izadi S., Sellen A., Perry M. (2007). Homes that make us smart. Pers. Ubiquitous Comput..

[5] Niyato D., Hossain E., Camorlinga S. (2009). Remote patient monitoring service using eterogeneous wireless access networks: Architecture and optimization. IEEE J. Sel. Areas Commun..

[6] Heer T., Garcia-Morchon O., Hummen R., Keoh S.L., Kumar S.S., Wehrle K. (2011). Security Challenges in the IP-based Internet of Things. Wirel. Pers. Commun..

[7] Roman R., Zhou J., Lopez J. (2013). On the features and challenges of security and privacy in distributed internet of things. Comput. Netw..

[8] Rescola E., Modadugu N. RFC 4347: Datagram Transport Layer Security (DTLS), 2006. https://tools.ietf.org/html/rfc4347.

[9] IETF Datagram Transport Layer Security for the Internet of Things (DTLS-IoT) Working Group. https://datatracker.ietf.org/wg/dice/charter/.

[10] IETF Authentication and Authorization for Constrained Environments (ACE) Working Group. https://datatracker.ietf.org/wg/ace/charter/.

[11] Piedra A.D.L., Braeken A., Touhafi A. (2013). Extending the IEEE 802.15.4 Security Suite with a Compact Implementation of the NIST P-192/B-163 Elliptic Curves. Sensors.

[12] Choi Y., Lee D., Kim J., Jung J., Nam J., Won D. (2014). Security Enhanced User Authentication Protocol for Wireless Sensor Networks Using Elliptic Curves Cryptography. Sensors.

[13] Nam J., Kim M., Paik J., Lee Y., Won D. (2014). A Provably-Secure ECC-Based Authentication Scheme for Wireless Sensor Networks. Sensors.

[14] Yeh H.L., Chen T.H., Liu P.C., Kim T.H., Wei H.W. (2011). A Secured Authentication Protocol for Wireless Sensor Networks Using Elliptic Curves Cryptography. Sensors.

[15] Pawlowski M.P., Jara A.J., Ogorzalek M.J. Extending Extensible Authentication Protocol over IEEE 802.15.4 networks. Proceedings of the 8th International Conference on Innovative Mobile and Internet Services in Ubiquitous Computing (IMIS-2014).

[16] Hernandez-Ramos J.L., Pawlowski M.P., Jara A.J., Skarmeta A.F., Ladid L. (2015). Towards a Lightweight Authentication and Authorization Framework for Smart Objects. IEEE J. Sel. Areas Commun..

[17] Marin L., Jara A.J., Gómez-Skarmeta A.F. (2013). Shifting primes: Optimizing elliptic curve cryptography for 16-bit devices without hardware multiplier. Math. Comput. Model..

[18] Edwards H.M. (2007). A normal form for elliptic curves. Bull. Am. Math. Soc..

[19] Bernstein D.J., Birkner P., Joye M., Lange T., Peters C. (2008). Twisted Edwards Curves. Lect. Notes Comput. Sci..

[20] Bernstein D., Lange T. Explicit Formula Database, 2007. https://www.hyperelliptic.org/EFD/.

[21] Hisil H., Wong K.K.H., Carter G., Dawson E. (2008). Twisted Edwards Curves Revisited. Lect. Notes Comput. Sci..

[22] Marin L. Differential Elliptic Point Addition in Twisted Edwards Curves. Proceedings of the 2013 27th International Conference on Advanced Information Networking and Applications Workshops (WAINA).

[23] Rivest R. The MD5 Message-Digest Algorithm, 1992. http://tools.ietf.org/tml/rfc1321?ref=driverlayer.com.

[24] Eastlake D., Jones P. US Secure Hash Algorithm 1 (SHA1), 2001. https://tools.ietf.org/html/rfc3174.

[25] Pub N.F. Secure Hash Standard. http://csrc.nist.gov/publications/fips/fips180-2/fips180-2withchangenotice.pdf.

[26] Jara A.J. (2014). Trust Extension Protocol for Authentication in Networks Oriented to Management (TEPANOM). Lect. Notes Comput. Sci..

